# Effect of lamotrigine in the treatment of bipolar depression with psychotic features: a case report

**DOI:** 10.1186/s12991-017-0154-2

**Published:** 2017-08-09

**Authors:** Tomoko Kajiya, Hiroko Sugawara, Yusuke Kajio, Satoru Morieda, Hibiki Tanaka, Tadashi Jono, Noboru Fujise, Mamoru Hashimoto

**Affiliations:** 1 Department of Neuropsychiatry, Faculty of Life Science, Kumamoto University, 1-1-1 Honjo, Chuo-ku, Kumamoto-Shi, Kumamoto 860-8556 Japan; 2 Kumamoto University Healthcare Center, Kumamoto, Japan

**Keywords:** Lamotrigine, Bipolar depression, Psychotic features, Mood-incongruent, Glutamatergic function

## Abstract

**Background:**

Major depressive episodes with psychotic features are more common in bipolar disorder than in major depressive disorder; however, there is little information on the optimal treatment for bipolar depression with psychotic features.

**Case presentation:**

The patient was a 69-year-old man. At the age of 66, he was admitted to the hospital for the treatment of bipolar depression with psychotic features. He was treated with a combination therapy of antipsychotics and antidepressants during long-term hospitalization. At the age of 69, he relapsed and was admitted to the hospital again. He was initially treated with olanzapine and lithium for the treatment of bipolar depression with psychotic features. He partially responded to the combination therapy, and psychomotor retardation and delusion of guilt disappeared; however, he developed psychomotor agitation and delusion of persecution, which was a mood-incongruent psychotic feature. Finally, he fully recovered with an additional dosage of lamotrigine, and had no experience of relapse after discontinuation of olanzapine.

**Conclusions:**

This case report implicates the utility of lamotrigine for bipolar depression with psychotic features, and further studies are needed to establish the optimal treatment.

## Background

Major depressive episodes with psychotic features are more common in bipolar disorder (BD) than in major depressive disorder (MDD) [1–3], and a recent study has reported that depressed bipolar patients with psychotic features have worse illness outcomes [4]. Most treatment guidelines have recommended either the combination of an antidepressant with an antipsychotic or electroconvulsive therapy (ECT) for the treatment of MDD with psychotic features [5, 6]; however, there is little information on the optimal treatment for bipolar depression with psychotic features.

Lamotrigine, which is known as an antiepileptic mood stabilizer, is a regulatory approved maintenance therapy for BD to prevent relapse, predominantly depressive episodes [7, 8]. There has been some uncertainty; however, the effect of lamotrigine monotherapy in the acute phase of bipolar depression has been reported in a previous meta-analysis [9].

Here, we report the case of a bipolar depression patient with psychotic features who was responsive to the addition of lamotrigine to lithium therapy.

## Case presentation

The patient was a 69-year-old man. He had no history of psychiatric disorder before the age of 47. He had a depressive episode at the age of 47, and fully recovered and maintained remission. At the age of 66, he became more talkative than usual and suddenly thought about starting an electronic enterprise while in an elevated mood, alongside increased activity and a decreased need for sleep. After his first hypomanic episode, he gradually became anxious and regretted his behavior in the hypomanic state. He finally developed delusions associated with a depressive mood, and was admitted to our department. He was diagnosed with bipolar depression with mood-congruent psychotic features. While he developed catatonia after the admission, an ECT was not performed due to the comorbidity of deep-vein thrombosis. He was treated with a combination therapy of various antipsychotics and antidepressants; however, he developed refractory depression, failing to respond to pharmacotherapy. It took considerable time for remission and he was discharged after 8 months of hospitalization. At the age of 69, he became depressive with delusions after the second hypomanic episode, and was admitted to our department again. He had a depressed mood, insomnia, appetite loss, psychomotor retardation, and inappropriate guilt, which was delusional. He was initially treated with olanzapine up to 17.5 mg/day (increasing 2.5–5.0 mg/day per 2 weeks), and given lithium up to 800 mg/day (increasing 100–200 mg/day per 2 weeks) for the treatment of bipolar depression with psychotic features. He partially responded to the combination therapy of lithium and olanzapine for about 2 months and psychomotor retardation and delusion of guilt disappeared; however, he developed psychomotor agitation and delusion of persecution, which was a mood-incongruent psychotic feature. Increasing the dosage of olanzapine to 17.5 mg/day caused over sedation, and was not effective for his mood-incongruent psychotic depression. Finally, lamotrigine was started, and psychomotor agitation and delusion of persecution gradually disappeared by increasing the dose up to 200 mg/day (increasing 25–50 mg/day per 2 weeks). He fully recovered taking lithium and lamotrigine for about 1 month, and had no experience of relapse after discontinuation of olanzapine.

## Discussion and conclusions

Most studies of psychotic symptoms in BD focus on manic episodes with psychotic features because psychotic symptoms are more common in manic episodes [10, 11], and there are some suggestions for manic episodes with psychotic features in a recent review of treatment guidelines for BD [12]. On the other hand, the prevalence of psychotic features in depressive episode could be higher in BD, especially bipolar I disorder, than MDD [1, 2]; however, there are few studies of pharmacotherapy for bipolar depression with psychotic features. A recent study, which investigated the differences in psychopharmacological treatment between psychotic and non-psychotic episodes in BD, reported that psychotic bipolar depression was associated with treatment with antipsychotics and the combination of an antipsychotic and an antidepressant [13]. As for the present case, he was treated with a combination therapy of antipsychotics and antidepressants on his first admission due to his first episode of bipolar depression with mood-congruent psychotic features. However, the efficacy of antidepressants for the treatment of bipolar depression has not been established [14, 15].

Lamotrigine, which blocks sodium channels and reduces synaptic glutamate release in the brain [16], has been considered to be a potential treatment for schizophrenia, as a large amount of evidence implicates the dysfunction of glutamatergic neurotransmission in the pathophysiology [17]. In fact, it has been shown that lamotrigine and clozapine may have a synergic effect in decreasing phencyclidine (PCP)-induced hyper locomotion [18], and the first meta-analysis has shown the efficacy of lamotrigine augmentation for clozapine-resistant schizophrenia patients [19]. Recently, it has been reported that lamotrigine reversed behavioral change in a neurodevelopmental animal model for schizophrenia combined with neonatal injection of PCP and post-weaning social isolation [20]. In the present case, there are possibilities of a natural course of remission as time passed, and the effect of long exposure to the combination therapy of lithium and olanzapine. However, the combination therapy was effective only for his depressive symptoms focused mainly on psychomotor retardation and delusion of guilt, not for psychomotor agitation and delusion of persecution. Besides, he responded to the addition of lamotrigine along with decreasing the dosage of olanzapine, and fully recovered despite the discontinuation of olanzapine. Lamotrigine may improve psychosis through the effect on glutamatergic function, and the pharmacological action is different from that of antipsychotics, whose main effect is as a dopamine antagonist. On the other hand, there are some reports of psychiatric side effects of lamotrigine, such as manic switches, acute psychotic episodes, and hallucinations [21], and the pharmacological action of lamotrigine for psychotic symptoms remains unclear.

This case report implicates the utility of lamotrigine for bipolar depression with psychotic features, and further studies are needed to establish the optimal treatment.

## Abbreviations

BD: bipolar disorder
MDD: major depressive disorder
ECT: electroconvulsive therapy
PCP: phencyclidine
